# Metallization and Diffusion Bonding of CoSb_3_-Based Thermoelectric Materials

**DOI:** 10.3390/ma13051130

**Published:** 2020-03-03

**Authors:** Hangbin Feng, Lixia Zhang, Jialun Zhang, Wenqin Gou, Sujuan Zhong, Guanxing Zhang, Huiyuan Geng, Jicai Feng

**Affiliations:** 1 State Key Laboratory of Advanced Welding and Joining, Harbin Institute of Technology, Harbin 150001, China; 18245017507@163.com (H.F.); zhangjialunhit@163.com (J.Z.); 19S009143@stu.hit.edu.cn (W.G.); fjc_hit@163.com (J.F.); 2 Zhengzhou Research Institute of Mechanical Engineering, Zhengzhou 450001, China; zsj_hit@163.com (S.Z.); zgx_hit1@163.com (G.Z.)

**Keywords:** thermoelectric materials, joining, skutterudite alloy, Co-Mo metallization layer

## Abstract

CoSb_3_-based skutterudite alloy is one of the most promising thermoelectric materials in the middle temperature range (room temperature—550 °C). However, the realization of an appropriate metallization layer directly on the sintered skutterudite pellet is indispensable for the real thermoelectric generation application. Here, we report an approach to prepare the metallization layer and the subsequent diffusion bonding method for the high-performance multi-filled *n*-type skutterudite alloys. Using the electroplating followed by low-temperature annealing approaches, we successfully fabricated a Co-Mo metallization layer on the surface of the skutterudite alloy. The coefficient of thermal expansion of the electroplated layer was optimized by changing its chemical composition, which can be controlled by the electroplating temperature, current and the concentration of the Mo ions in the solution. We then joined the metallized skutterudite leg to the Cu-Mo electrode using a diffusion bonding method performed at 600 °C and 1 MPa for 10 min. The Co-Mo/skutterudite interfaces exhibit extremely low specific contact resistivity of 1.41 μΩ cm^2^. The metallization layer inhibited the elemental inter-diffusion to less than 11 µm after annealing at 550 °C for 60 h, indicating a good thermal stability. The current results pave the way for the large-scale fabrication of CoSb_3_-based thermoelectric modules.

## 1. Introduction

Thermoelectric materials have attracted great attention for centuries because they can directly convert heat to electricity, and vice versa [1]. Nowadays, thermoelectric generation is also an important green energy technology for solving the energy crisis [2]. Among many thermoelectric materials, the skutterudite (SKU) compound is one of the most promising thermoelectric materials for the middle temperature range of thermoelectric generation. The skutterudite compound not only possesses high thermoelectric performance, but also good mechanical properties and excellent economic and environmental friendliness [3,4,5,6,7,8]. 

In the fabrication of a thermoelectric device, both *p*-type and *n*-type thermoelectric materials are required to be connected with the metal electrodes [9]. However, due to problems such as the large contact resistance, elemental diffusion and coefficient of thermal expansion (CTE) mismatch, how to connect the skutterudites to the electrodes reliably is still a major challenge [10]. Copper and nickel are the common electrode materials for the thermoelectric devices. However, when the electrode connects to the skutterudite compounds directly, they react violently. This reaction results in a great degradation of the thermoelectric performance of the skutterudite compounds [11,12]. Therefore, it is indispensable to prepare a diffusion barrier layer on the surface of the skutterudite compound. At present, most of the diffusion barrier layers of thermoelectric materials are fabricated by the sintering method [13,14,15]. The connection is achieved using the brazing method [16]. For example, Guo et al. used Fe-Co-Ni alloys as the diffusion barriers suitable for their *p*- and *n*-type skutterudite compounds [16]. Co, Fe, Ni and some minor elements were arc melted into buttons. Round disks were then sliced from the buttons and sintered on the top and bottom sides of the *n*- and *p*-type pellets to serve as diffusion barriers. After that, the metallized pellets were brazed to the Cu electrodes using the Ag-Cu-Zn solders [16,17,18]. Gu et al. used the Ti-Al mixed powders as the diffusion barrier. The Ti-Al mixed powders were put on the top of the Yb_0.3_Co_4_Sb_12_ fine powders during the bonding process of the spark plasma sintering (SPS); the Ti powders reacted with Sb and Co to form a thin layer of intermetallic compound, which provided necessary bonding strength [19]. However, when the diffusion barrier layers were sintered with the thermoelectric powders, both the sintering temperature and pressure were limited by the physical properties of the thermoelectric materials; the barrier layer was often porous, resulting in the large contact resistivity of the thermoelectric leg. Furthermore, the preparation of the barrier layer by the sintering method often resulted in the difficulty of controlling the size of the thermoelectric leg accurately. The size of the sintered pellets was also limited by the thermal stresses caused by the CTE mismatch. Thus, these features were inconvenient for the large-scale device fabrication. 

Electroplating metallic layers on thermoelectric materials followed by annealing is widely used in the thermoelectric community [20]. It has been reported that a cobalt barrier layer was prepared on the surface of a skutterudite leg by the electroplating method. However, since the CTE of cobalt is larger than that of the skutterudite alloy, the joint was easy to crack [21]. It was also reported that a Ti barrier layer on the surface of the skutterudite alloy was prepared by the magnetron sputtering approach [22]. The CTE of Ti was similar to that of the skutterudite alloy; but the diffusion ability of Ti in the skutterudite alloy was very weak, resulting in low bonding strength between them [23]. Zhao et al. prepared a Ti barrier layer by the SPS method and systematically studied the diffusion kinetics of the Ti/CoSb_3_ interface at high temperatures. Using the interfacial shearing strength as the criteria, Zhao et al. also predicted a service life of over 20 years for the Ti/CoSb_3_ joints at 500 °C [24,25]. 

It is clear that the realization of an appropriate diffusion barrier layer directly on the sintered skutterudite pellet is critical for the real thermoelectric generation application. Here, we first synthesized multifilled *n*-type skutterudite pellets. Then, the Co-Mo nanograined layers were fabricated on the surface of the pellets by the traditional electroplating and subsequent low-temperature annealing approaches. Both the CTE and the elemental diffusion behaviors were controlled by tuning the Mo content in the layer. Finally, a reliable joint between the skutterudite alloy and the Cu-Mo electrode was achieved using the diffusion bonding method.

## 2. Materials and Methods 

### 2.1. Fabrication of the Skutterudite Alloys

Yb ingot (99.9%, Alfa Aesar), Al ingot (99.9%, Alfa Aesar), Ga ingot (99.99%, Alfa Aesar), In ingot (99.9%, Alfa Aesar), Co ingot (99.95%, Alfa Aesar), Ca granules (99.5%, Alfa Aesar), Fe granules (99.98%, Alfa Aesar) and Sb balls (99.999%, Alfa Aesar, Ward Hill, US) were weighted according to the chemical composition of Yb_0.3_Ca_0.1_Al_0.1_Ga_0.1_In_0.1_Co_4_Sb_12_. The weighted elements were loaded into a carbon crucible and then were sealed in the quartz tubes under vacuum below 10^−3^ Pa. The quartz tubes were slowly heated to 1150 °C, held at this temperature for 5 h and then quenched in cold water. The quenched ingots were annealed at 700 °C for 150 h. The annealed samples were ground into fine powders in sizes of < 75 μm in a glovebox filled with high-purity Ar. Finally, the powders were sintered using SPS (Dr. Sinter Lab, Tsurugashima, Japan) at 700 °C for 15 min under a pressure of 60 MPa. 

### 2.2. The Electroplating Process

The bulk skutterudite pellets were cut into cubes in the size of 5 mm × 5 mm × 5 mm using a diamond wire saw (STX-202A, Shenyang, China). The surfaces of the cubes were then polished. Before electroplating, the cubes were first placed in acetone for ultrasonic cleaning for 10 min to remove the oil stain on the surface of the material. During the plating of Co-Mo alloy on the surface of the skutterudite cubes, the temperature of the plating solution was set to 30–60 °C; the pH of the plating solution was controlled at 5.0–7.0 and the current was set to 0.07–0.16 A. It took about 30 min for the electroplating process. The plated skutterudite cubes were then sealed in the quartz tubes under vacuum below 10^−3^ Pa. Thereafter, the quartz tubes were placed in a box furnace for annealing at 450 °C for 2 h. Finally, the metallized skutterudite cubes were diffusion bonded to the Cu-Mo electrode. The applied pressure was about 1 MPa, and the joints were kept at 600 °C for 10 min to complete the diffusion bonding. To study the thermal stability of the metallization layer, we further annealed the joint at 550 °C in vacuum for 2 h, 5 h, 50 h, and 60 h, respectively. 

### 2.3. Characterizations 

Electrical transport properties of the obtained skutterudite alloys, including electrical conductivity and the Seebeck coefficient, were measured using the CTA-3 (Cryoall Co. Ltd, Beijing, China) apparatus. The maximum *ZT* of the *n*-type skutterudite compound was about 1.35@773K. The details of the thermoelectric properties of the compound will be reported in our future manuscripts. The interfacial microstructure of the joints was investigated by a scanning electron microscope (Quanta 200FEG and Merlin Compact, Thermo Fisher Scientific, Waltham, US), and the chemical composition of the interfacial diffusion layers was analyzed using an energy dispersive spectrometer (EDS). The specific contact resistivity of the joints was measured on homemade four-probe equipment. The uncertainty of the homemade equipment is 5%.

## 3. Results and Discussion

### 3.1. Metallization of Skutterudite Alloys

Since the skutterudite-based thermoelectric generators usually work between 50 °C and 600 °C, it is necessary to prepare a barrier layer on the surface of the skutterudite. Otherwise, the Cu-based electrodes will react violently with the skutterudite leg, leading to the lower joint strength and thermoelectric performance of the generator. Generally speaking, the choice of the barrier layer needs to meet the following criteria: (i) The barrier layer can simultaneously form a finite reaction layer with the skutterudite compound and the electrode to form a high-strength joint. (ii) The barrier layer should possess a similar CTE to that of the skutterudite compound and the metal electrode. (iii) The interfacial electrical and thermal resistances caused by the introduction of the barrier layer should be as low as possible. According to the above criteria, the Co-Mo alloy was chosen as the barrier layer between the skutterudite compound and the Cu-Mo electrodes. 

**Figure 1:**
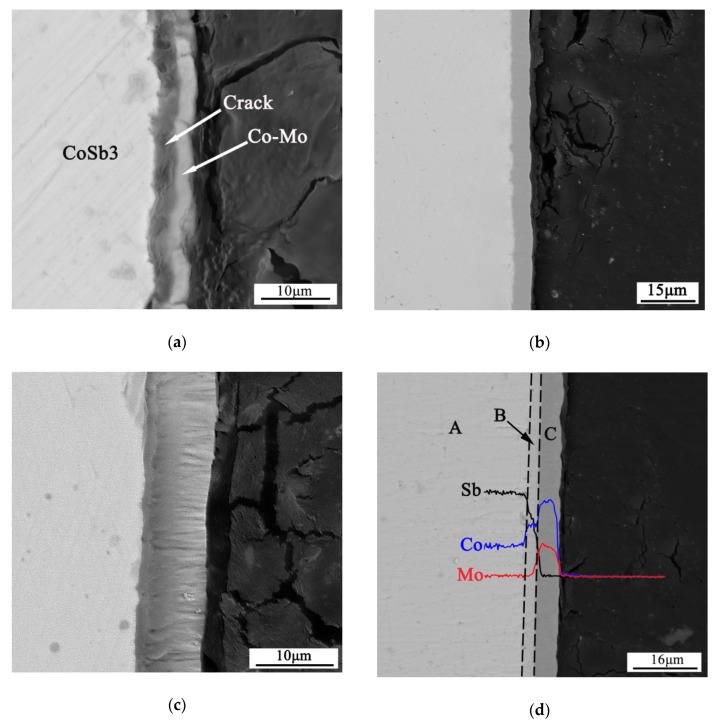
The backscattered electron (BSE) micrograph of Co-Mo/skutterudite (SKU) couple: (**a**) Before annealing, some parts of the electroplated barrier layer were separated from the skutterudite. (**b**) Annealed at 450 °C for 2 h, a metallurgical bonding between the barrier layer and the skutterudite compound was achieved. (**c**) The columnar crystals of the Co-Mo barrier layer. (**d**) The Co-Mo/SKU couple reacted at 500 °C for 5h. CoSb_2_ of 3 μm was formed between the electroplated layer and skutterudite compound.

Figure 1a shows the cross-section of the electroplated layer. It is clear that the electroplated layer was just mechanically bonded to the surface of the skutterudite compound. Some parts of the electroplated barrier layer were separated from the skutterudite just after the electroplating process. The bonding strength between the barrier layer and the skutterudite alloy was far from the requirement of an ideal connection. To ensure the bonding strength, subsequent annealing under vacuum was carried out. As shown in Figure 1b, after annealing at 450 °C for 2 h, a metallurgical bonding between the barrier layer and the skutterudite compound was achieved. Figure 1c shows the high-magnification backscattered electron (BSE) cross-section image of the interface between the skutterudite alloy and the electroplated layer. A thin reaction layer in a thickness of 1 μm formed just on the top of the skutterudite compound. Some nanograined columnar crystals inside the barrier layer can also be observed clearly. The nanostructures in the electroplated layer introduced a vast amount of highly defective grain boundaries, which greatly reduced the diffusion energy barrier in the electroplated layer. Thus, a metallurgical bonding could be achieved at the low annealing temperature of 450 °C.

To gain insight into the reaction mechanism of the interfacial layer after annealing, we further increased the annealing temperature and time to obtain a thicker reaction layer. Figure 1d shows the cross-section microstructure and the EDS line-scanning results of the interface between the skutterudite compound and the electroplated layer after annealing at 500 °C for 5 h. According to the EDS results listed in Table 1, the Sb atoms diffused into the electroplated layer and CoSb_2_ was formed between the electroplated layer and the skutterudite compound. The thickness of the reaction layer increased to about 3 μm.

### 3.2. Tuning the CTE of the Barrier Layer

**Figure 2:**
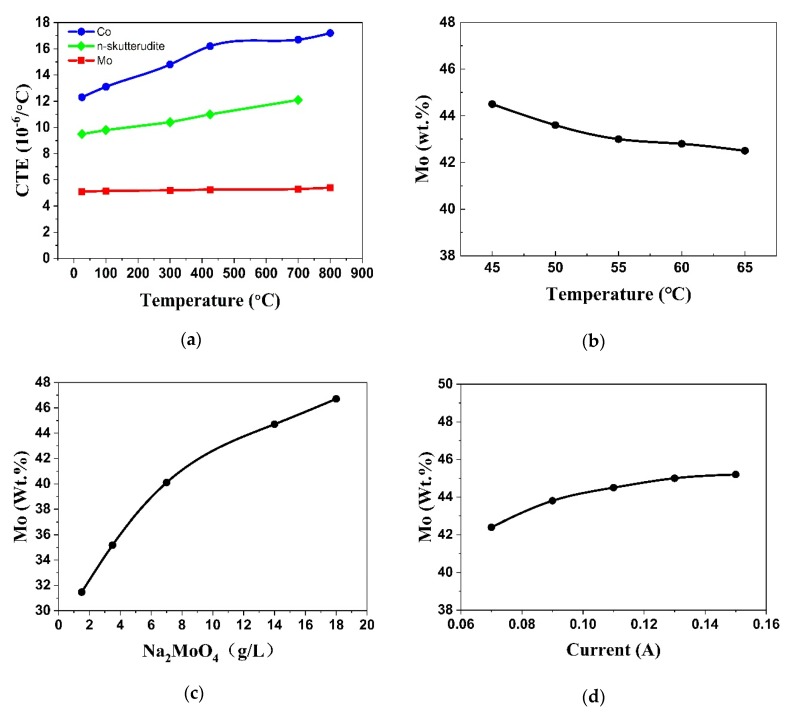
(**a**) The temperature-dependent coefficient of thermal expansion (CTE) of Co, skutterudite compound and Mo. (**b**) The effects of temperature on the Mo content of the electroplated layer. (**c**) The effects of the Na_2_MoO_4_ concentration on the Mo content of the electroplated layer. (**d**) The effects of the current on the Mo content of the electroplated layer.

To reduce the thermal stresses between the thermoelectric materials and the electrode, the diffusion barrier should possess CTE close to those of the skutterudite compounds from room temperature to 600 °C. Guo et al. pointed out that the mismatch ratio of their CTEs should be limited to less than 10% [16]. The CTE of skutterudite is difficult to change, but the CTEs of Co-Mo alloys can be easily controlled by adjusting the chemical composition, as shown in Figure 2a. In fact, Song et al. demonstrated that the Co_0.6_Mo_0.4_ powder-mixed composite can serve well as a diffusion barrier for their *n*-type skutterudite compounds [26].

Our results indicate that the Mo content in the Co-Mo barrier layer can be adjusted by changing the conditions of plating such as the temperature, current and composition of the plating solution. Figure 2b shows that when the current is 0.08 A and the content of Na_2_MoO_4_ in the plating solution is 8 g/L, the Mo concentration decreases slightly with the increasing temperature. On the contrary, when the current is 0.08A and the temperature is 65 °C, the content of Mo in the electroplated layer increases remarkably as the concentration of Na_2_MoO_4_ in the plating solution increases (Figure 2c). As for the current, the Mo content in the electroplated layer increases slightly with the increasing current when the temperature is about 65 °C and the content of Na_2_MoO_4_ in the plating solution is 8 g/L (as shown in Figure 2d).

**Figure 3:**
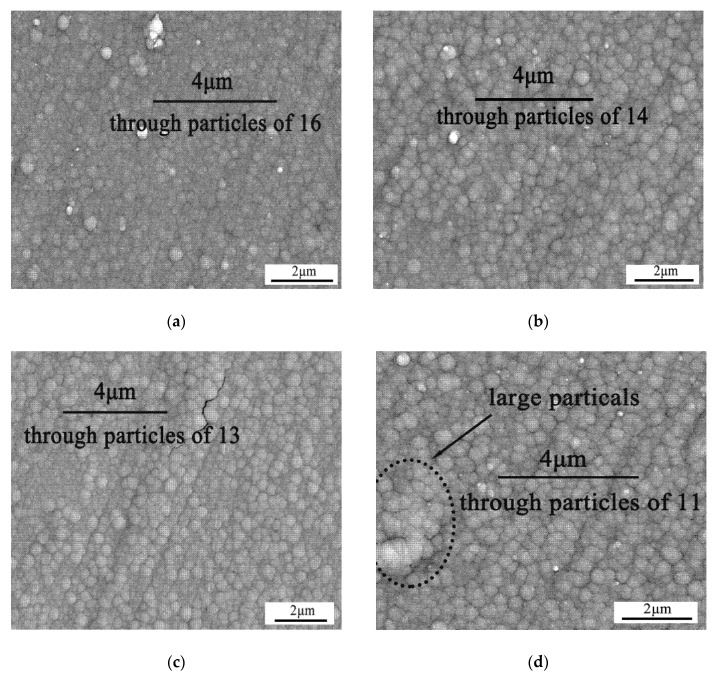
The BSE micrographs of the as-electroplated Co-Mo barrier layers with current of: (**a**) 0.09 A, (**b**) 0.11 A, (**c**) 0.13 A and (**d**) 0.15 A. The grain size of the electroplated layer increases gradually with the increasing current.

The current can affect not only the composition of the electroplated layer, but also the surface morphology of the electroplated layer. As shown in Figure 3, the surface of the Co-Mo electroplated layer contains many nanosized particles. As the current increases, some cracks appear in the surface, and the particle size gradually increases. The large particles will reduce the number of highly-defective grain boundaries and decrease the activity of the electroplated layer. The reason is that the metal deposition rate increases with the increasing current. When the current is less than 0.09 A, the deposition process of Co and Mo keeps stable. According to the above results, the most efficient method to adjust the composition and the CTE of the Co-Mo electroplated layer is to change the content of Na_2_MoO_4_ in the plating solution.

**Figure 4:**
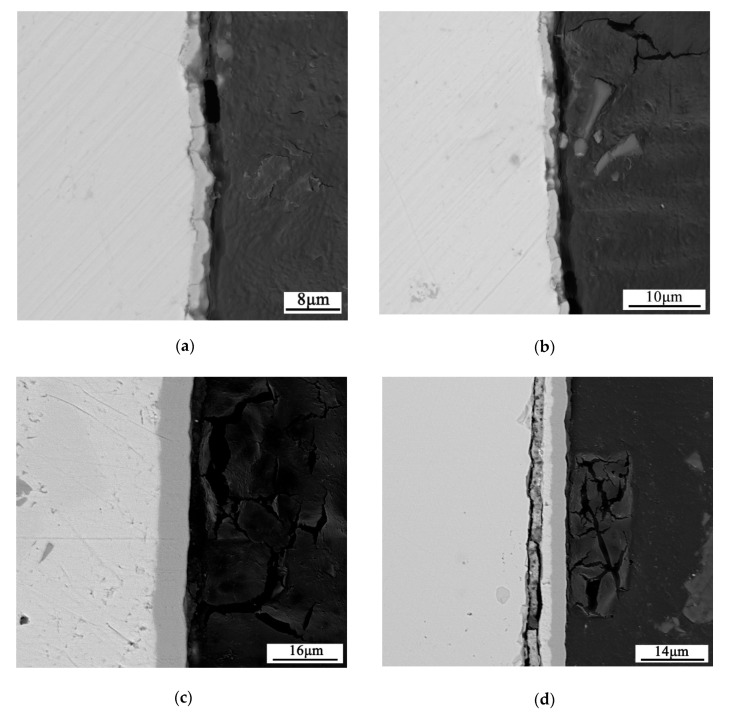
The cross-sectional BSE micrograph of the Co-x wt % Mo/SKU interface: (**a**) x = 50, (**b**) x = 40, (**c**) x = 35, (**d**) x = 25. No cracks were found either in the annealed layer or the skutterudite compound when x = 35.

Figure 4 shows the electroplated layers after annealing at 450 °C for 2 h. For the layers with Mo content greater than 40 wt %, cracks appear in the annealed layer (Figure 4a,b), indicating that the CTE of the layer is less than that of the skutterudite compound. On the other hand, for the layers with Mo content less than 30 wt %, cracks appear in the skutterudite compound (Figure 4d), indicating that the CTE of the layer is greater than that of the skutterudite compound. Figure 4c shows the layer with Mo content of 30 wt %–40 wt %. No cracks are found either in the annealed layer or in the skutterudite compound. We have extended the current method to the *p*-type skutterudite. The preliminary results show that a metallurgical bonding of Co-Mo to the skutterudite compound can also be achieved. Since the CTE of *p*-type skutterudite is larger than that of *n*-type skutterudite, the chemical composition of the Co-Mo layer should be further optimized. The details will be reported in our future manuscripts.

### 3.3. Diffusion Bonding between the Skutterudite Compound and the Cu-Mo Electrode

**Figure 5:**
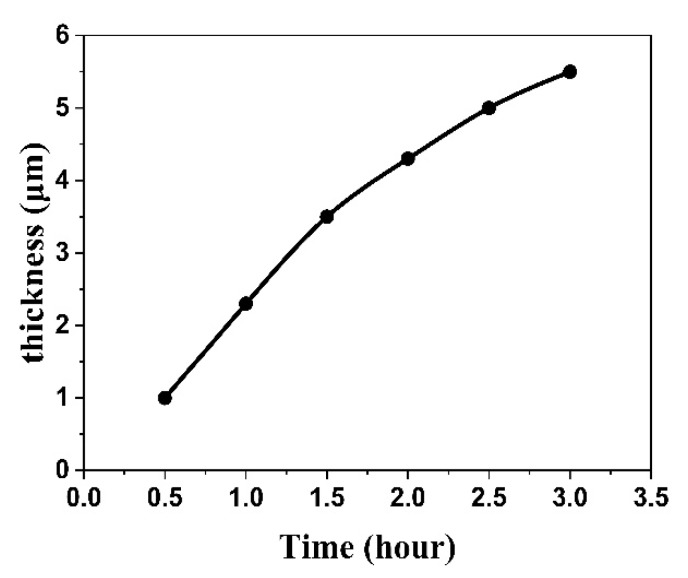
The effects of plating time on the thickness of the electroplated layer.

To join the skutterudite compound to the Cu-Mo electrode, we first prepared a thick Co-Mo electroplated layer on the surface of the skutterudite compound. As shown in Figure 5, the thickness of the layer increases with the increasing electroplating time. The growth rate of the Co-Mo barrier layer is about 2 μm/h.

**Figure 6:**
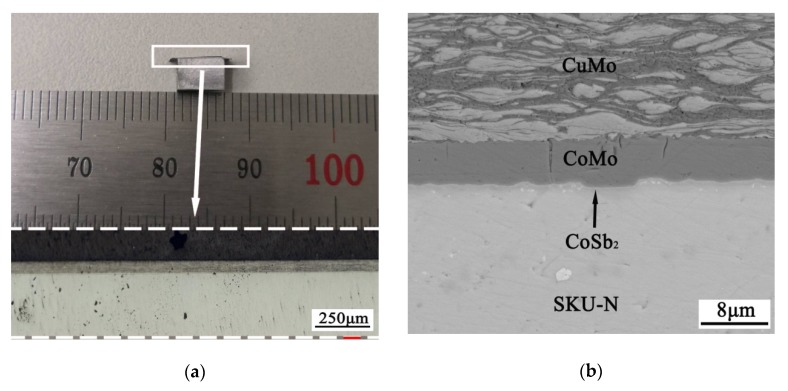
(**a**) The diffusion bonded joint between the skutterudite compound and the Cu-Mo electrode. (**b**) The BSE image of the cross-section of the joint. A reaction layer (CoSb_2_) of 2–3 μm forms between the skutterudite compound and the Co-Mo layer after the diffusion bonding process.

Figure 6a shows a typical joint of the skutterudite compound metallized by Co-Mo and the Cu-Mo electrode. The joint is fabricated by the diffusion bonding method at 600 °C and 1 MPa for 10 min. A reaction layer, CoSb_2_ of 2–3 μm, formed between the skutterudite compound and the Co-Mo layer (as shown in Figure 6b). On the contrary, there is no obvious reaction layer between the Co-Mo layer and the Cu-Mo electrode.

**Figure 7:**
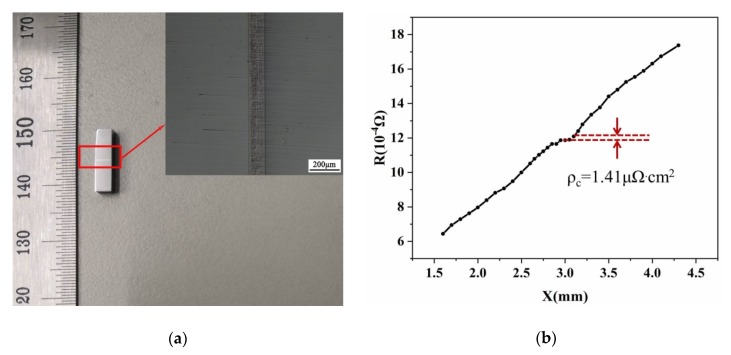
(**a**) The skutterudite–electrode–skutterudite sandwich structure fabricated by the diffusion bonding method. (**b**) The measured specific contact resistivity of the Co-Mo/skutterudite interface.

To investigate the specific contact resistivity (SCR) of the joint, we also fabricate a skutterudite–electrode–skutterudite sandwich structure (as shown in Figure 7a) using the same technology as described above. The SCR is, thus, measured on this sandwich structure using our homemade instrument. The room-temperature electrical resistivity of the skutterudite alloy is measured by the homemade instrument, too. The value of 2.85 × 10^−6^ Ω m is consistent with the CTA-3 result, indicating the reliability of our homemade equipment. 

The Co-Mo/SKU interfaces fabricated by the current work exhibit extremely low SCR of 1.41 μΩ·cm^2^ (as shown in Figure 7b). This value is among one of the lowest SCRs to date reported for the thermoelectric modules as shown in Table 2. 

**Figure 8:**
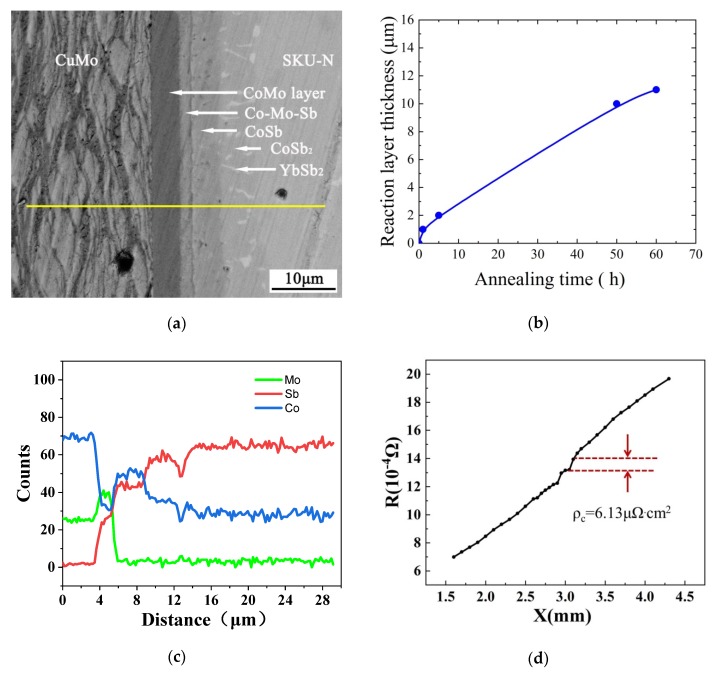
(**a**) The SEM image of the CuMo/CoMo/SKU diffusion bonded joint after annealing at 550 °C for 60 h. About 11 μm reaction layer of Co-Mo-Sb/CoSb/CoSb_2_ is formed. (**b**) The diffusion layer thickness vs. annealing time for the CuMo/CoMo/SKU joint. (**c**) The EDS line-scan profiles of the CuMo/CoMo/SKU diffusion bonded joint after annealing at 550 °C for 60 h. (**d**) The specific contact resistivity (SCR) of 6.13 μΩ.cm^2^ of the CuMo/CoMo/SKU joint after annealing at 550 °C for 60 h.

Figure 8a shows the microstructure of the CuMo/CoMo/SKU interface after annealing at 550 °C for 60 h. According to the EDS line-scan results (as shown in Figure 8c), the reaction layer mainly contains three parts: the Co-Sb-Mo layer, the CoSb layer and the CoSb_2_ layer. Compared with the joint before annealing (as shown in Figure 6b), a CoSb layer (~6 μm in thickness) appears between the CoMo and CoSb_2_ layers. The thickness of the CoSb2 layer increases to about 5 μm. The atomic concentration of Mo increases remarkably in the Co-Sb-Mo layer. Therefore, it can be expected that the enrichment of Mo will suppress the diffusion of Sb by blocking the diffusion path [29]. In fact, Figure 8b shows the evolution of the thickness of the reaction layer of the CoMo/SKU interface as a function of annealing time. It can be seen that the thickness of the diffusion layer is only about 11 μm after annealing at 550 °C for 60 h. As the growth thickness (*L*) of the diffusion layer is linear with the square root of the annealing time (t), it is expressed by the following equation [26]:

(1) $$L=a+b\sqrt{t}$$

where *a* and *b* are fitting parameters. The best fitting shows that *a* = 3.7 μm, *b* = 0.92 μm/h^1/2^. According to the fitting results, the thickness of the diffusion layer after one-year annealing is predicted to be about 90 μm.

Figure 8d shows the measured SCR of the CuMo/CoMo/SKU joint after annealing at 550 °C for 60 h. The SCR of 6.13 μΩ·cm^2^ is slightly higher than that of the as-bonded one. This value is still among the lowest ones according to Table 2. Since the contact resistivity is somehow proportional to the diffusion layer thickness, we can roughly estimate the contact resistivity after one-year annealing is about 50 μΩ·cm^2^. Both the EDS line-scan and the SCR results demonstrate that the metallization layer fabricated in the current work is thermally stable.

We have tried to measure the shear strength of the joint before and after the long-term annealing. The fractures always appear inside the skutterudite leg instead of the joint itself. This result indicates that the diffusion layer will not weaken the mechanical strength of the joint.

## 4. Conclusions

We introduce an approach to prepare the metallization layer and the subsequent diffusion bonding method for the high-performance multifilled *n*-type skutterudite compounds. Using the electroplating followed by the low-temperature annealing approaches, we successfully fabricated a Co-Mo metallization layer on the surface of the skutterudite compound. The CTE of the electroplated layer can be optimized by changing the weight ratio between Co and Mo, which is controlled by the electroplating temperature, current and the concentration of the Mo ions in the solution. We also find that the most efficient method to adjust the composition of the Co-Mo layer is to change the concentration of Na_2_MoO_4_ in the plating solution. We then joined the metallized skutterudite leg to the Cu-Mo electrode using a diffusion bonding method performed at 600 °C and 1 MPa for 10 min. The Co-Mo/SKU interfaces exhibit extremely low specific contact resistivity of 1.41 μΩ·cm^2^. The metallization layer fabricated in the current work is thermally stable after annealing at 550 °C for 60 h.

## Figures and Tables

**Table 1:** Analysis of interface components (at %).

	Co	Sb	Mo	Possible Phase
A	26.10	73.87	0.03	CoSb_3_
B	33.01	66.93	0.06	CoSb_2_
C	74.90	0.25	24.85	Co-Mo intermetallic phases

**Table 2:** The specific contact resistivity for various classes of thermoelectric materials.

Thermoelectric Materials	Metallization Layer	Specific Contact Resistivity (μΩ·cm^2^)
Yb_0.3_Co_4_Sb_12_ [14]	Mo-Ti	9
Bi_2_Te_3_ [27]	Au	2.73
Mg_2_Si [28]	Ni	210
Hf_0.5_Zr_0.5_CoSn_0.2_Sb_0.8_ [29]	Ag	38
(Mm,Sm)yCo_4_Sb_12_ [26]	Fe-Ni	2
n-type skutterudite [30]	CoSi_2_	0.4
This work	Co-Mo	1.41
